# Relationship between Mid-Upper Arm Circumference and Body Mass Index in Inpatients

**DOI:** 10.1371/journal.pone.0160480

**Published:** 2016-08-05

**Authors:** Néstor Benítez Brito, José Pablo Suárez Llanos, Manuel Fuentes Ferrer, Jose Gregorio Oliva García, Irina Delgado Brito, Francisca Pereyra-García Castro, Nieves Caracena Castellanos, Candelaria Xiomara Acevedo Rodríguez, Enrique Palacio Abizanda

**Affiliations:** 1 Endocrinology and Nutrition Department of Hospital Universitario Nuestra Señora de Candelaria, Ctra. Del Rosario n°145, 38010, Santa Cruz de Tenerife, Spain; 2 Preventitive Medicine Department of Hospital Clínico San Carlos, Calle Profesor Martín Lagos, S/N, 28040, Madrid, Spain; Hospital Universitario de la Princesa, SPAIN

## Abstract

**Introduction:**

Nutritional screening is a fundamental aspect of the initial evaluation of the hospitalised patient. Body Mass Index (BMI) in association with other parameters is a good marker of malnutrition (<18.5 kg/m^2^), but it presents the handicap that the great majority of patients cannot be weighed and measured. For this reason it is necessary to find other indicators that can be measured in these patients.

**Objectives:**

1) Analyse the relationship between BMI and Mid-Upper Arm Circumference (MUAC); 2) establish a cut-off point of MUAC equivalent to BMI <18.5 kg/m^2^.

**Materials and Methods:**

The anthropometric data of patients hospitalised over the period 2004–2013 were retrospectively revised. The following variables were collected: weight, height, BMI, MUAC, sex and age.

**Results:**

1373 patients were evaluated, who presented a mean weight of: 65.04±15.51 kg; height: 1.66±0.09 m; BMI: 23.48±5.03 kg/m^2^; MUAC: 26.95±4.50 cm; age: 56.24±16.77. MUAC correlates suitably to BMI by means of the following equation (simple linear regression): BMI = − 0.042 + 0.873 x MUAC (cm) (R^2^ = 0.609), with a Pearson r value of 0.78 (p<0.001). The area under the curve of MUAC for the diagnosis of malnutrition was 0.92 (95% CI: 0.90–0.94; p<0.001). The MUAC value ≤22.5 cm presented a sensitivity of 67.7%, specificity of 94.5%, and a correct classification of 90%. No significant statistical differences were found in the cut-off point of MUAC for the diagnosis of malnutrition based on sex (p = 0.115) and age (p = 0.694).

**Conclusions:**

1) MUAC correlates positively and significantly with BMI. 2) MUAC ≤ 22.5 cm correlates properly with a BMI of <18.5 kg/m^2^, independent of the age or sex of the patient, although there are other alternatives. MUAC constitutes a useful tool as a marker of malnutrition, fundamentally in patients for whom weight and height cannot be determined.

## Introduction

Hospital Malnutrition (HM) is highly prevalent. It is estimated that around 30 million people in Europe present HM, and furthermore, it is associated with elevated costs of some €170 billion annually [1]. In this sense, a risk of malnutrition of 32.6% was observed in the EuroOOPS Study [2], in which 5,061 patients admitted to European hospitals were evaluated. HM is associated with delay in patient recovery, prolongs hospital stays, increases the number of readmissions, increases the risk of infection, alters the quality of life and increases mortality [3–6].

Body Mass Index (BMI) corresponds to weight in kg/height in m^2^, and is a good marker of malnutrition, present in the majority of existing nutritional screens. However, it must be taken into account that many inpatients cannot be weighed and measured. Consequently, there is a need for markers easier to measure to detect malnutrition in habitual clinical practice. Currently, a BMI of 18.5 kg/m^2^ is considered a marker of malnutrition when associated with other risk factors, although, per se, this is not of much predictive value as it does not exclude patients with constitutional underweight [7,8].

Mid-Upper Arm Circumference (MUAC) is a simple measurement which has been used for many years in nutritional evaluation, being an indicator of protein and energy reserves of the individual. Diverse studies have employed MUAC as a nutritional parameter in different population groups (as elderly, inpatients, infants, pre-school-age children, schoolchildren, pregnant women or lactating women) [9–16].

In every day hospital practice, it is often impossible to weigh or measure the patient, thus frustrating BMI calculation. In this case, estimated weight and height can be obtained through indirect measures that have been demonstrated to correlate with direct measures. In this sense, the height could be measured in an indirect way through the length of the arm and the height of the knee, whereas the estimated weight is more difficult to calculate, because this requires several anthropometric values, and larger deviations from real weight can occur. These indirect measures undeniably lead to an inherent margin of error in the process of estimating height or weight, being more relevant in the case of height as the latter is squared in the BMI equation. For this reason, another anthropometric measurement that relates to BMI would have to be used. MUAC could be one, especially as several studies have examined this association [9,10,17].

During the design on the part of our team of a hospital nutritional screen termed CIPA (Control of food Intake, Proteins and Anthropometry)[18–19] that incorporates as an item BMI <18.5 kg/m^2^, one of the major problems observed was that a large part of the patients could not be weighed or measured. For instance, certain pathologies or conditions impeded correct height measurement (kyphosis, vertebral compression fracture due to osteoporosis, scoliosis, flexor weakness), while others hampered determination of body weight (inability to stand) or might even have affected real weight (pregnancy, oedema, ascites). Therefore, BMI was calculated by means of methods which diminish the reliability of the test (e.g., estimated weight, habitual weight or height, according to the patient).

Through this study, MUAC will be studied to determine whether it can be a useful parameter to categorise the nutritional state of the patient for whom BMI cannot be measured. To this end a relationship between MUAC and BMI in inpatients has been observed, and concretely, an adequate cut-off point for MUAC that correlates to a BMI of <18.5 kg/m^2^.

## Materials and Methods

A retrospective study was carried out of 1,373 patients assessed by the Clinical and Dietetic Nutrition Section of Hospital Universitario Nuestra Señora de Candelaria (HUNSC) in the period comprising 2004 to 2013.

The ethics committee of HUNSC gave it's approval for the carrying out of this study on the date 27 March 2014. Given that this was a retrospective study of patients screened by the clinical and dietetic nutrition section, it was not possible to obtain informed consent due to the peculiarities of the study. The patient’s information was anonymized and de-identified prior to analysis.

All inpatients were included whatever their basic pathology, and who had received a nutritional assessment by Nutrition Department staff with anthropometric data (weight, height, BMI, MUAC). All patients with oedema in the extremities or ascites, absence of any limb, as well as patients with data missed in the anthropometric assessment were excluded. The following data were collected: age, sex, weight, height, BMI and MUAC.

To obtain MUAC a flexible, inelastic measuring tape model Seca 201 was used, and the measure was taken in the patient's non-dominant arm, just at the mid-point between the acromion and the olecranon, in sitting or standing posture. The value obtained was expressed in centimetres. MUAC was taken twice and the averages were recorded. Weight and height were obtained with a model Seca 220 scale, placing the patient with their back to the instrument, measuring the height and weight. BMI was calculated by analysis software using the formula: weight (kg)/ height (m)^2^.

### Statistical analysis

The qualitative variables are presented with their frequency distributions. The quantitative variables are summarised by mean and standard deviation (SD). The linear relationship between MUAC and BMI was studied via the calculation of the Pearson linear correlation coefficient and the estimation of the simple linear regression coefficients of the equation. The determination coefficient (R2) is calculated as a goodness-of-fit index. For the comparisons of the quantitative variables between two independent groups the Student's parametric *t*-test is used. An analysis of covariance (ANCOVA) was carried out with the aim of evaluating the effect of sex and the presence of malnutrition based on BMI on the averages values of MUAC. For the continuous variable MUAC a Receiver Operating Characteristic (ROC) curve was plotted with the aim of obtaining a global measure of the accuracy of the test for the combination of all the possible cut-off points. The area under the curve (AUC) was calculated along with its confidence interval (CI) to 95%. Based on the coordinates of the curve, a cut-off point is selected for the variable MUAC, and the sensitivity, specificity, likelihood ratio for positive and negative test, as well as the percentage of correct classification were carried out. A logistic regression model is adjusted with the aim of evaluating, using the interaction term whether the selected cut-off point presented a different effect from the classification of malnutrition based on sex and age. Null hypothesis was rejected by a type I error minor than 0.05. The statistical analysis was carried out using the STATA statistics package, version 12.0.

## Results

The parameters of 1,373 patients (43.3% women) were collected, whose initial data can be found listed by subgroup in Table 1. 16.9% of the sample presented a BMI <18.5 kg/m^2^.

**Table 1:** Anthropometric characteristics of the sample.

Variable	Overall (mean± SD)	Men (n = 778) (mean± SD)	Women (n = 595) (mean± SD)	p*
**Weight (kg)**	65.04±15.51	69.49±15.08	59.21±14.07	<0.001
**Height (m)**	1.66±0.09	1.71±0,07	1.59±0.07	0.001
**BMI (kg/m**^**2**^**)**	23.48±5.03	23.71±4.83	23.17±5.25	0.048
**MUAC (cm)**	26.95±4.49	27.47±4.24	26.26±4.72	0.001
**Age (years)**	56.24±16.77	56.94±16.25	55.32±17.39	0.078

* Statistical Significance Student’s t-Test;

Kilogram (Kg); Metre (m); Centimetre (cm); Standard Deviation (SD).

**Fig 1:**
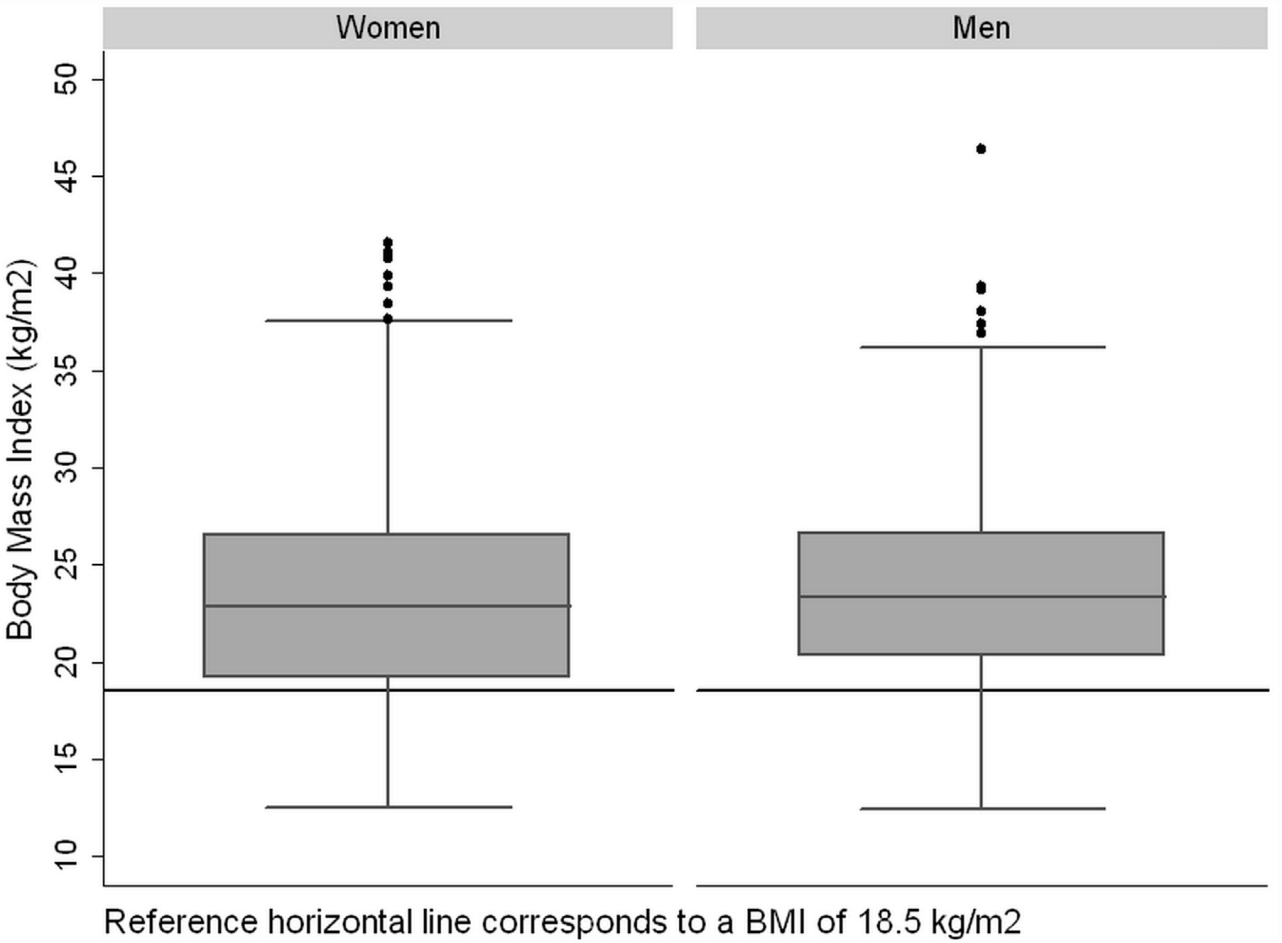
Box plot of Body Mass Index (kg/m^2^) in women and men.

Regarding sex, men presented in absolute terms an average MUAC 1.2 cm (95% CI, 0.73–1.69) greater than women (p<0.001). As for BMI, the distribution is different between men and women (quantifying the risk of malnutrition as BMI <18.5 kg/m^2^), being statistically greater in women (20.2%) than in men (14.3%) (p = 0.024) (Fig 1).

**Fig 2:**
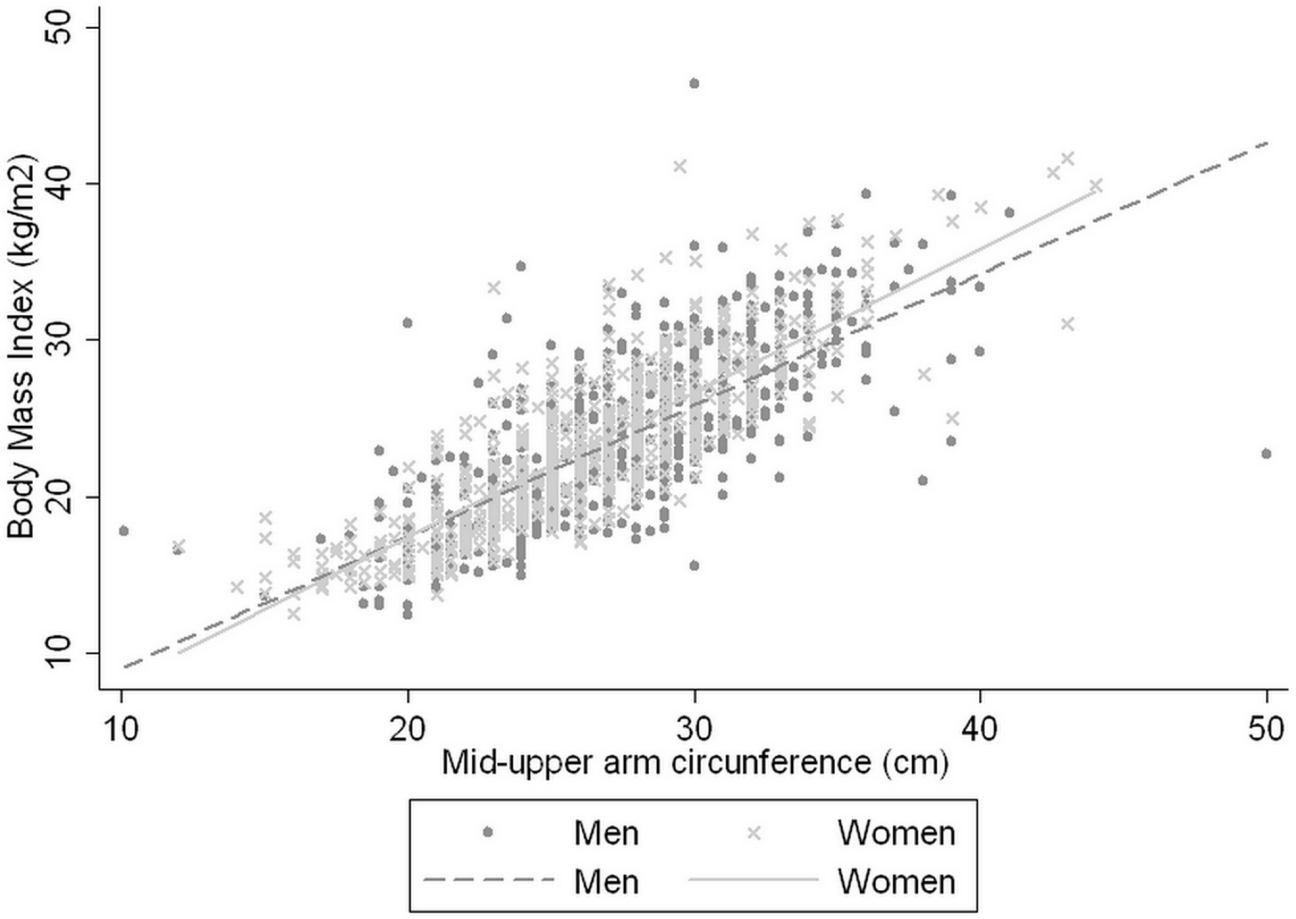
Relationship between Mid-Upper Arm Circumference and Body Mass Index in women and men.

MUAC shows a significant relationship with BMI by means of the equation BMI = −0.042 + 0.873 x MUAC (cm) (R2 = 0.609), with a Pearson correlation coefficient of 0.78 (95% CI: 0.76–0.80), while that in men was of 0.83 (95% CI: 0.80–0.85) and in women 0.74 (95% CI: 0.70–0.77), as set out in Fig 2.

The mean MUAC of the group of patients with malnutrition (21.4 (3.1)) was statistically inferior (p<0.001) to the group of patients without malnutrition (28.1 (3.9)). When stratified by sex, malnourished women presented in absolute terms a mean MUAC of 7.04 cm less (95% CI: 6.2–7.7; p<0.001), while in men the difference was 6.05 (95% CI: 5.31–6.79; p<0.001). Based on the ANCOVA results no statistically significant interaction was observed (p = 0.064) between sex and the presence of malnutrition in relation to MUAC.

**Fig 3:**
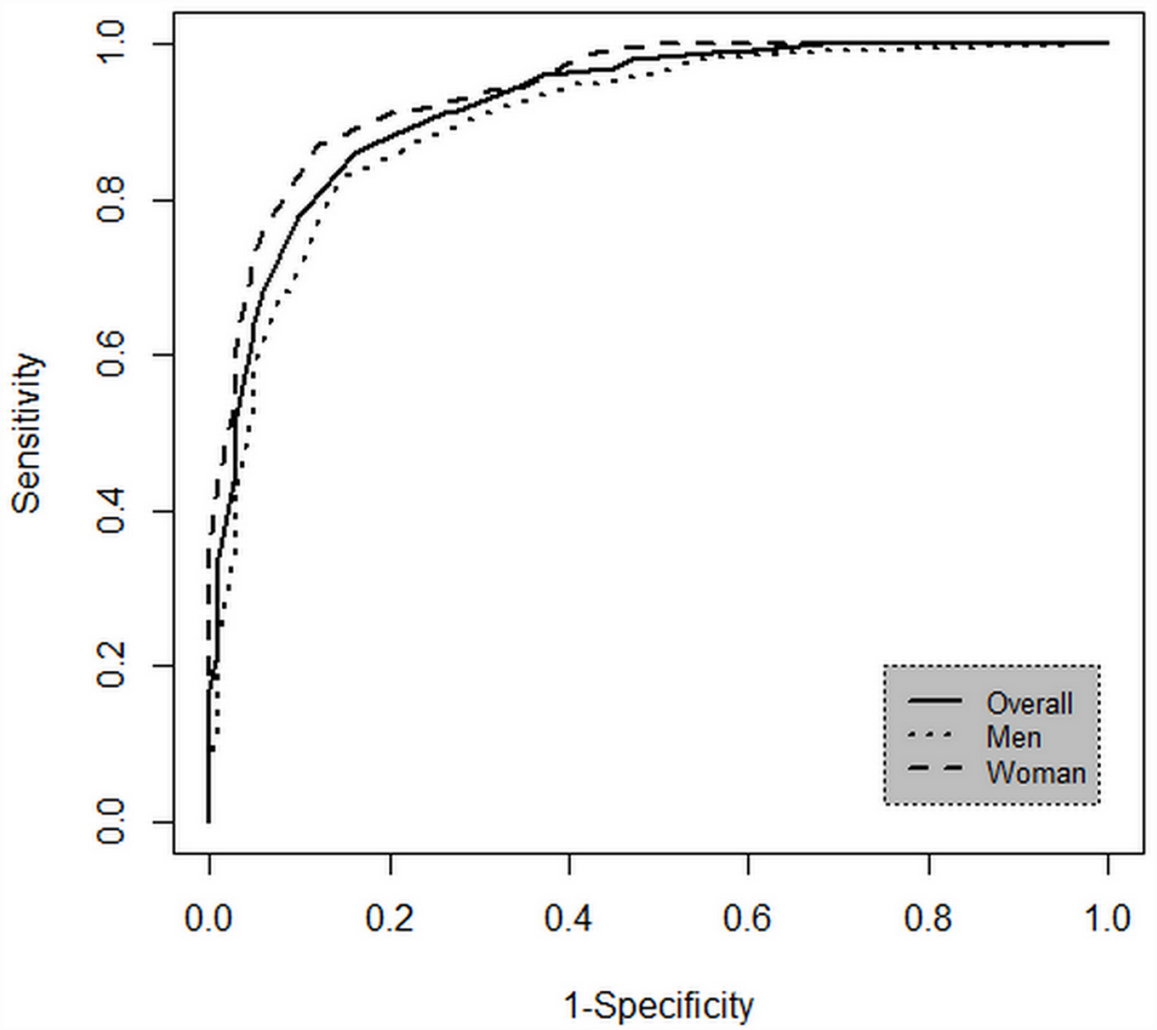
Area Under the Curve of Mid-Upper Arm Circunference for the detection of malnutrition in the whole sample and stratified by sex.

The AUC of MUAC for the detection of malnutrition in the whole sample was 0.92 (95% CI: 0.90–094; p<0.001). The results in women and men were as follows: AUC: 0.94 (95% CI: 0.92–0.96; p<0.001) and AUC: 0.91 (95% CI: 0.88–0.93; p<0.001), respectively (Fig 3). Based on the coordinates of the curve, cut-off points for the whole of the sample were selected by the values of correct classification. Table 2 shows the index of sensitivity, specificity, likelihood ratio for positive and negative test for different values of MUAC.

**Table 2:** Diagnostic validity of Mid-Upper Arm Circunference for the detection of malnutricion (Body Mass Index <18,5 kg/m^2^).

Cut-off points for MUAC (cm)	≤24.0	≤23.5	≤23.0	≤22.5	≤22.0	≤21.5	≤21.0
**Sensitivity (%)**	85.78	78.88	77.59	67.67	62.93	52.16	45.26
**Specificity (%)**	84.37	89.20	90.34	94.47	95.00	96.58	96.84
**Likelihood ratio for positive test**	5.49	7.30	8.03	12.23	12.58	15.23	14.32
**Likelihood ratio for negative test**	0.17	0.24	0.25	0.34	0.39	0.50	0.57
**Correct classification (%)**	84.7	87.46	88.2	90.0	89.57	89.1	88.11

According to the results of the logistic regression models, the selected cut-off point for MUAC did not show statistically significant differences in relation to sex (p = 0.115) or age (p = 0.694).

## Discussion

The results of the present study suggest that MUAC serves as a predictor of BMI. Likewise, the selected cut-off point of ≤22.5 cm presents a high percentage of correct classifications (BMI <18.5 kg/m^2^).

In everyday clinical practice we encounter a serious inconvenience when we try to carry out a nutritional assessment on a patient who cannot be measured or weighed. The use of subjective parameters such as those provided by the patient, or estimated weight and height, are habitual in clinical practice. Eriksen J. et al. [20] demonstrated that the patient's perception of weight loss is usually incorrect, thus reducing effectiveness.

There are more than a few papers that have studied this relationship. Indeed of the fewer studies there are in adults, there are even fewer the ones carried out in developed countries [21].

The Nutrition Risk Screening 2002 (NRS 2002) [17] advised that there was little evidence in existence to translate values of BMI values to the corresponding MUAC. Nevertheless, the investigators by undertaking a retrospective analysis of various studies, indicate that an MUAC of <25 cm may correspond to a BMI of <20.5 kg/m^2^.

On the other hand, Malnutrition Universal Screening Tool (MUST) [22] suggests that a MUAC less than 23.5 cm is probably associated with a BMI less than 20 kg/m^2^, and thus the patient is found to be in a state of risk of malnutrition. This study also deals with weight loss over time and values of MUAC. In this respect they estimated that if MUAC changed by at least 10%, weight and BMI would probably also change by 10% or more.

In turn, the Mini Nutritional Assessment (MNA) screening, designated particularly for a population over 65 years of age, also comprises MUAC data, including values of 22–21 cm as parameters to detect malnutrition [23].

Sultana T et al. [9] recently published a study similar to ours, but by means of a prospective analysis of 650 patients who attended a hospital in Bangladesh with a profile of more acute patients. In that study, even though the sample was separated by sex, they also found a strong correlation between MUAC and BMI with some indices of correlation and an AUC similar to those found in our study.

The same paper also attempted to categorise some levels of MUAC that might correspond to a BMI of <18.5 kg/m^2^, the authors concluding that a MUAC <25 cm is identified with said BMI in males, and <24 cm in females. It should be taken into account, however, the differences that exist between the study population of this work and that of ours, not only for the interracial variability that could justify the differences encountered, but also for the greater chronic profile of our patients, for instance greater average age, which is almost double. This is probably a reflection of the important socioeconomic differences between the two countries, for which reason our work may be more representative of general hospitals in first-world countries. For this reason, people over 60 years of age were not excluded from our work (as was done in the study by Sultana et al,[9]) since life expectancy is elevated, and therefore these patients are a sample of the real characteristics of inpatients in this type of hospital.

A retrospective study, also with patients suffering from acute pathology, identified a good correlation between BMI and MUAC, and even the patients with a MUAC <25 cm presented a greater length of stay during hospitalisation and a higher incidence of mortality[24]. In this sense, MUAC also proved to be a good predictor of mortality in the elderly population of Taiwan, even better than BMI[25]. Nguyen P et al.[10], for their part, also undertook a retrospective study in Vietnam in which they found a good correlation between MUAC and BMI in healthy pre-menopausal women, a MUAC of <23.5 cm being in this case the value that best corresponded to a BMI of <18.5 kg/m^2^.

The sample of patients in our work was not only large but also very heterogeneous, scattered between the medical as well as surgical departments of a general hospital, thus reflecting the characteristics of usual clinical practice. The study was performed in Western Europe, understanding that the results would not coincide with the majority of studies carried out in developing countries due to interracial differences in body composition.

We identified that there is a good correlation between MUAC and BMI, just like that occurring in the work of Sultana et al [9], as much in the global sample as in the sex-separated analysis. Therefore, MUAC adequately predicts values of BMI.

To facilitate the evaluation of the results obtained by measuring MUAC, we sought to analyse the results in a grouped sample of men and women. Thus, it was found that the differences in MUAC between malnourished and non-malnourished patients were somewhat greater in women than in men, but without reaching statistical significance, being able to employ said common value to both sexes in the analysis of this measure. Furthermore, to be able to generalise the threshold of MUAC that represents a BMI of <18.5 kg/m^2^, the relationship of the results to patient age was studied, finding that a value ≤22.5 cm is applicable to any inpatient.

Through this analysis, and observing that the possible cut-off points of each sex are very close to one another, the conclusion was reached that it is impractical to use a different value for men and women. It gains nothing in terms of classification capacity, and moreover, the intention in our case is to introduce the measure into a hospital nutritional screen where a high number of patients will be subjected to it. For this reason the greatest simplicity possible must be sought with optimum results. Understanding that the classification of BMI is the same for women as for men, it is reasoned that there is no clinical justification for it being different for MUAC, based on the observations in this sample.

Our team specifically chose the cut-off point of MUAC ≤22.5 cm based on the results found in Table 2, to include it in the CIPA screen in conjunction with other nutritional parameters, independently of the sex and age of the patient. This cut-off point is centred on obtaining a low percentage of misclassified patients, maintaining an acceptable sensitivity and specificity. The selection of a higher cut-off point, increasing sensitivity, involved an increase in false positive and a reduction in correct classification.

It is possible that to use MUAC in a different setting, other authors choose a higher cut-off point which gives greater sensitivity, or to choose various cut-off points according to sex or age, but we think that for its inclusion in a nutrition screening tool that facilitates to the maximum possible degree the functions of healthcare personnel, a single common cut-off point must be used. Moreover, based on the reduced muscle mass, it may be more convenient to choose a lower cut-off point in an elderly population. However, our analyses did not reveal significant differences for subjects aged over 65 using the selected cut-off point. With the value we have chosen of ≤22.5 cm included in the CIPA screen, we have been able to to predict a worse clinical prognosis in those patients that test positive, increasing mortality more than four-fold and hospital stay by a week (in contrast to the results obtained with the Subjective Global Assessment)[26].

Our work reveals various limitations which should be explained. Firstly, it deals with a retrospective study with the attendant limitations that involves, albeit with a significant number of patients. As we have commented on in the discussion, different cut-off points can be obtained according to sex and age, but the objective of this study was to find a single common cut-off point for a nutritional screen.

We believe, in any case, that the data obtained in our study are significant, since it is the first to be undertaken with a sample of inpatients from a general hospital in the first world. Furthermore, in Spain as well as in Europe there is a body of work in combating malnutrition related to illness in which many inpatient nutritional screens are been designed and applied, and so we believe that the results obtained in our article contribute somewhat more objectivity to the anthropometric data included in those studies.

In conclusion, this work authenticates MUAC as a parameter that correlates very well with BMI and which can be used as a complement to it, or as a substitute for the same in situations in which the patient cannot be weighed or measured. Furthermore, different cut-off points for MUAC are presented which correlate with BMI <18.5 kg/m^2^ (global as well as differentiated by sex) that provide different sensitivities, specificities, and correct classification, so that, depending on use and context, can serve in one or another value. We believe that for the introduction of this variable into a hospital nutritional screen, it would be ideal for said cut-off point to be common to both sexes and independent of patient age, to facilitate the task of those performing the nutritional screen, a key aspect of its implementation in a general hospital setting.

## Abbreviations

BMI: Body Mass Index
MUAC: Mid-Upper Arm Circumference
HM: Hospital Malnutrition
CIPA: Control of food Intake, Proteins and Anthropometry
CI: Confidence Interval
AUC: Area Under the Curve
HUNSC: Hospital Universitario Nuestra Señora de Candelaria

## References

[1] Ljungqvist O, Man FD. Under nutrition: a major health problem in Europe. Nutr Hosp. 2009; 24(3): 369–370. 19721916

[2] Sorensen J, Kondrup J, Prokopowicz J, Schiesser M, Krähenbühl L, Meier R, et al EuroOOPS study group. EuroOOPS: an international, multicentre study to implement nutritional risk screening and evaluate clinical outcome. Clin Nutr, 2008; 27(3): 340–49. 10.1016/j.clnu.2008.03.012 18504063

[3] Correia MI, Waitzberg DL. The impact of malnutrition on morbidity, mortality, length of hospital stay and costs evaluated through a multivariate model analysis. Clin Nutr. 2003; 22(3): 235–9. 12765661 10.1016/s0261-5614(02)00215-7

[4] Reilly JJ Jr, Hull SF, Albert N, Walker A, Bringardener S. Economic impact of malnutrition: a model system for hospitalized patients. JPEN. 1988; 12(4): 371–6. 10.1177/0148607188012004371 3138447

[5] Bickford GR, Brugler LJ, Dolsen S, Vickery CE. Nutrition assessment outcomes: a strategy to improve healthcare. Clin Lab Manage Rev. 1999; 13(6): 357–64. 10747662

[6] Perez de la Cruz A, Lobo Támer G, Orduña Espinosa R, Mellado Pastor C, Aguayo de Hoyos E, Ruiz López MD. Malnutrition in hospitalized patients: prevalence and economic impact. Med Clin (Barc). 2004; 10; 123(6) 201–6. 15282072 10.1016/s0025-7753(04)74461-9

[7] Cederholm T, Bosaeus T, Barazzoni R, Bauer J, Van Gossum A, Klek S, et al Diagnostic criteria for malnutrition—An ESPEN Consensus Statement. Clin Nutr. 2015; 34(3): 335–40. 10.1016/j.clnu.2015.03.001 25799486

[8] Álvarez J, Del Río J, Planas M, García Peris P, García de Lorenzo A, Calvo V, et al SENPE’s document group. SENPE-SEDOM document on coding of hospital hyponutrition. Nutr Hosp. 2008; 23(6): 536–40. 19132260

[9] Sultana T, Karim MN, Ahmed T, Hossain MI. Assessment of under nutrition of Bangladeshi adults using anthropometry: can body mass index be replaced by mid-upper-arm-circumference? PLoS One. 2015; 10(4): e0121456 10.1371/journal.pone.0121456 25875397 PMC4397021

[10] Nguyen P, Ramakrishnan U, Katz B, González-Casanova I, Lowe AE, Nguyen H, et al Mid-upper-arm and calf circumferences are useful predictors of underweight in women of reproductive age in northern Vietnam. Food Nutr Bull. 2014; 35(3): 301–11. 25902590 10.1177/156482651403500303

[11] Khadivzadeh T. Mid upper arm and calf circunferences as indicators of nutritional status in women of reproductive age. East Mediterr Health J. 2002; 8(4–5); 612–8. 15603044

[12] Chakraborty R, Bose K, Koziel S. Use of mid-upper arm circunference in determining undernutrition and illness in rural adult Oraon men of Gumla District, Jharkhand, India. Rural Remote Health. 2011; 11(3): 1754 21882889

[13] Mayor MC, Guerrero-Segundo M. Correlation of anthropometry with the presence of malnutrition in the elderly. Rev Sanid Milit Mex. 2012; 66(1): 17–28.

[14] Aparecida Leandro-Merhi V, Luiz Braga de Aquino J, Gonzaga Teixeira de Camargo J. Agreement between body mass index, calf circumference, arm circumference, habitual energy intake and the MNA in hospitalized elderly. J Nutr Health Aging. 2012; 16(2): 128–32. 22323346 10.1007/s12603-011-0098-1 PMC12878115

[15] Corvos Hidalgo C. Anthropometric assessment of nutritional status using the circumference of the arm in university students. Nutr Clín Diet Hosp. 2011; 31(3): 22–27.

[16] Gueresi P, Miglio R, Cevenini E, Gualdi Russo E. Arm measurements as determinants of further survival in centenarians. Exp Gerontol. 2014; 58-230–4. 10.1016/j.exger.2014.08.012. 25172624

[17] Kondrup J, Rasmussen HH, Hamberg O, Stanga Z. Nutritional risk screening (NRS 2002): a new method based on an analysis of controlled clinical trials. Clin Nutr. 2003; 22(3): 321–36. 12765673 10.1016/s0261-5614(02)00214-5

[18] Oliva García JG, Pereyra-García Castro F, Benítez Brito N, Herrera Rodríguez EM, Suárez Llanos JP, García Bray BF, et al Validation of a method of dispensing nutritional supplements in a tertiary hospital. Nutr Hosp. 2013; 28(4): 1286–90. 10.3305/nh.2013.28.4.6479 23889654

[19] Suárez Llanos JP, Benítez Brito N, Oliva García JG, Pereyra-García Castro F, López Frías MA, García Hernández A, et al Introducing a mixed nutritional screening tool (CIPA) in a tertiary hospital. Nutr Hosp. 2014; 29(5): 1149–53. 10.3305/nh.2014.29.5.7299 24951997

[20] Eriksen J, Sneftrup SB. Anamnestic weight loss-do patients remember correctly? Eur J Clin Nutr. 2013; 67(6): 607–9. 10.1038/ejcn.2013.65 23511855

[21] Tang AM, Dong K, Deitchler M, Chung M, Maalouf-Manasseh Z, Tumilowicz A, et al 2013 Use of Cutoffs for Mid-Upper Arm Circumference (MUAC) as an Indicator or Predictor of Nutritional and Health-Related Outcomes in Adolescents and Adults: A Systematic Review. Washington, DC: FHI 360/FANTA.

[22] Sttratton RJ, Hackston A, Longmore D, Dixon R, Price S, Stroud M, et al Malnutrition in hospital outpatients and inpatients: prevalence, concurrent validity and ease of use of the “malnutrition universal screening tool” (MUST) for adults. Br J Nutr. 2004; 92(5): 799–808. 15533269 10.1079/bjn20041258

[23] Vellas B, Guigoz Y, Garry PJ, Nourahashemi F, Bennahum D, Lauque S, et al The Mini Nutritional Assessment (MNA) and Its Use in Grading the Nutritional State of Elderly Patients. Nutrition. 1999 2; 15(2): 116–22. 9990575 10.1016/s0899-9007(98)00171-3

[24] Powell-Tuck J, Hennessy EM. A comparison of mid upper arm circumference, body mass index and weight loss as indices of undernutrition in acutely hospitalized patients. Clin Nutr. 2003; 22(3): 307–12. 12765671 10.1016/s0261-5614(03)00009-8

[25] Tsai AC, Chang TL. The effectiveness of BMI, calf circumference and mid-arm circumference in predicting subsequent mortality risk in elderly Taiwanese. Br J Nutr. 2011; 105(2): 275–81. 10.1017/S0007114510003429 21129232

[26] Mora Mendoza A, Benítez Brito N, Suárez Llanos JP, Delgado Brito MI, Pérez Méndez LI, Oliva García JG, et al Validation of the nutritional screening CIPA in non-surgical pathologies compared with Subjective Global Assessment (SGA). P30. Congreso Nacional SENPE XXX. Nutr Hosp. 2015; (Supl.4) 31:1–144.

